# Supplementary material for: Tolerogenic dendritic cell reporting: Has a minimum information model made a difference?
Source: PeerJ. 2023 May 31;11:e15352. doi: 10.7717/peerj.15352 (PMC10239229; doi:10.7717/peerj.15352)
Supplement: Supplemental Information 1 [file peerj-11-15352-s001.docx]

**RAW DATA**

**References of Figure 5:**

1. Kumuthini, J., Mbiyavanga, M., Chimusa, E. R., Pathak, J., Somervuo, P., Van Schaik, R. H., ... & Squassina, A. (2016). Minimum information required for a DMET experiment reporting. *Pharmacogenomics*, *17*(14), 1533-1545. DOI: [10.2217/pgs-2016-0015](https://doi.org/10.2217/pgs-2016-0015).
2. Sakurai, K., Kurtz, A., Stacey, G., Sheldon, M., & Fujibuchi, W. (2016). First proposal of minimum information about a cellular assay for regenerative medicine. *Stem cells translational medicine*, *5*(10), 1345-1361. DOI: [10.5966/sctm.2015-0393](https://doi.org/10.5966/sctm.2015-0393).
3. Scudamore, C. L., Soilleux, E. J., Karp, N. A., Smith, K., Poulsom, R., Herrington, C. S., ... & Arends, M. J. (2016). Recommendations for minimum information for publication of experimental pathology data: MINPEPA guidelines. *The Journal of pathology*, *238*(2), 359-367. DOI: [**10.1002/path.4642**](https://doi.org/10.1002/path.4642).
4. Struwe, W. B., Agravat, S., Aoki-Kinoshita, K. F., Campbell, M. P., Costello, C. E., Dell, A., ... & Kettner, C. (2016). The minimum information required for a glycomics experiment (MIRAGE) project: sample preparation guidelines for reliable reporting of glycomics datasets. *Glycobiology*, *26*(9), 907-910. DOI: [10.1093/glycob/cww082](https://doi.org/10.1093/glycob/cww082).
5. Merino-Martinez, R., Norlin, L., van Enckevort, D., Anton, G., Schuffenhauer, S., Silander, K., ... & Litton, J. E. (2016). Toward global biobank integration by implementation of the minimum information about biobank data sharing (MIABIS 2.0 Core). *Biopreservation and biobanking*, *14*(4), 298-306. DOI: [10.1089/bio.2015.0070](https://doi.org/10.1089/bio.2015.0070).

**10 MITAP-compliant papers:**

- Spiering R, Jansen MA, Wood MJ, Fath AA, Eltherington O, Anderson AE, Pratt AG, Van Eden W, Isaacs JD, Broere F, Hilkens CM 2019 Targeting of tolerogenic dendritic cells to heat-shock proteins in inflammatory arthritis. Journal of translational medicine. 2019 Dec;17(1):1-2. DOI: [10.1186/s12967-019-2128-4](https://doi.org/10.1186/s12967-019-2128-4).
- Tomić S, Joksimović B, Bekić M, Vasiljević M, Milanović M, Čolić M, Vučević D 2019 Prostaglanin-E2 potentiates the suppressive functions of human mononuclear myeloid-derived suppressor cells and increases their capacity to expand IL-10-producing regulatory T cell subsets. Frontiers in immunology. 2019 Mar 18;10:475. DOI: [10.3389/fimmu.2019.00475](https://doi.org/10.3389/fimmu.2019.00475).
- Zubizarreta I, Flórez-Grau G, Vila G, Cabezón R, España C, Andorra M, Saiz A, Llufriu S, Sepulveda M, Sola-Valls N, Martinez-Lapiscina EH 2019 Immune tolerance in multiple sclerosis and neuromyelitis optica with peptide-loaded tolerogenic dendritic cells in a phase 1b trial. Proceedings of the National Academy of Sciences. 2019 Apr 23;116(17):8463-70. DOI: [10.1073/pnas.1820039116](https://www.pnas.org/cgi/doi/10.1073/pnas.1820039116).
- Anderson AE, Swan DJ, Wong OY, Buck M, Eltherington O, Harry RA, Patterson AM, Pratt AG, Reynolds G, Doran JP, Kirby JA 2017 Tolerogenic dendritic cells generated with dexamethasone and vitamin D3 regulate rheumatoid arthritis CD4+ T cells partly via transforming growth factor-β 1. Clinical & Experimental Immunology. 2017 Jan;187(1):113-23. DOI: [10.1111/cei.12870](https://doi.org/10.1111/cei.12870).
- Bouchet-Delbos L, Even A, Varey E, Saïagh S, Bercegeay S, Braudeau C, Dréno B, Blancho G, Josien R, Cuturi MC, Moreau A 2021 Preclinical assessment of autologous tolerogenic dendritic cells from end-stage renal disease patients. Transplantation. 2021 Apr 1;105(4):832-41. DOI: [10.1097/tp.0000000000003315.](https://doi.org/10.1097/tp.0000000000003315)
- Švajger, U., & Rožman, P. J. 2019 Synergistic effects of interferon-γ and vitamin D3 signaling in induction of ILT-3highPDL-1high tolerogenic dendritic cells. *Frontiers in immunology*, *10*, 2627. DOI: [10.3389/fimmu.2019.02627](https://doi.org/10.3389/fimmu.2019.02627).
- Ilic N, Gruden-Movsesijan A, Cvetkovic J, Tomic S, Vucevic DB, Aranzamendi C, Colic M, Pinelli E, Sofronic-Milosavljevic L 2018 Trichinella spiralis excretory–secretory products induce tolerogenic properties in human dendritic cells via Toll-like receptors 2 and 4. Frontiers in immunology. 2018 Jan 24;9:11. DOI: [10.3389/fimmu.2018.00011](https://doi.org/10.3389/fimmu.2018.00011).
- Funda DP, Goliáš J, Hudcovic T, Kozáková H, Špíšek R, Palová-Jelínková L 2018 Antigen loading (eg, glutamic acid decarboxylase 65) of tolerogenic DCs (tolDCs) reduces their capacity to prevent diabetes in the non-obese diabetes (NOD)-severe combined immunodeficiency model of adoptive cotransfer of diabetes as well as in NOD mice. Frontiers in immunology. 2018 Feb 16;9:290. DOI: [10.3389/fimmu.2018.00290](https://doi.org/10.3389/fimmu.2018.00290).
- García-González PA, Schinnerling K, Sepulveda-Gutierrez A, Maggi J, Hoyos L, Morales RA, Mehdi AM, Nel HJ, Soto L, Pesce B, Molina MC 2016 Treatment with dexamethasone and monophosphoryl lipid A removes disease-associated transcriptional signatures in monocyte-derived dendritic cells from rheumatoid arthritis patients and confers tolerogenic features. Frontiers in immunology. 2016 Oct 25;7:458. DOI: [10.3389/fimmu.2016.00458](https://doi.org/10.3389/fimmu.2016.00458).
- Hutchinson JA, Ahrens N, Geissler EK 2017 MITAP‐compliant characterization of human regulatory macrophages. Transplant International. 2017 Aug;30(8):765-75. DOI: [10.1111/tri.12988](https://doi.org/10.1111/tri.12988).

**10 Non-MITAP-compliant papers:**

- Garcia AM, Bishop EL, Li D, Jeffery LE, Garten A, Thakker A, Certo M, Mauro C, Tennant DA, Dimeloe S, Evelo CT 2021 Tolerogenic effects of 1, 25-dihydroxyvitamin D on dendritic cells involve induction of fatty acid synthesis. The Journal of steroid biochemistry and molecular biology. 2021 Jul 1;211:105891. DOI: [10.1016/j.jsbmb.2021.105891](https://doi.org/10.1016/j.jsbmb.2021.105891).
- Song HY, Kim WS, Han JM, Park WY, Lim ST, Byun EB. HMOC, a chrysin derivative, induces tolerogenic properties in lipopolysaccharide-stimulated dendritic cells. International Immunopharmacology. 2021 Jun 1;95:107523. DOI: [10.1016/j.intimp.2021.107523](https://doi.org/10.1016/j.intimp.2021.107523).
- Dawicki W, Huang H, Ma Y, Town J, Zhang X, Rudulier CD, Gordon JR 2021 CD40 signaling augments IL-10 expression and the tolerogenicity of IL-10-induced regulatory dendritic cells. PloS one. 2021 Apr 1;16(4):e0248290. DOI: [10.1371/journal.pone.0248290](https://doi.org/10.1371/journal.pone.0248290).
- Navarro-Barriuso J, Mansilla MJ, Quirant-Sánchez B, Teniente-Serra A, Ramo-Tello C, Martínez-Cáceres EM. Vitamin D3-induced tolerogenic dendritic cells modulate the transcriptomic profile of T CD4+ cells towards a functional hyporesponsiveness. Frontiers in immunology. 2021:3461. DOI: [10.3389/fimmu.2020.599623](https://doi.org/10.3389/fimmu.2020.599623).
- Lee HY, Kim J, Ryu JS, Park SJ. Trichomonas vaginalis α-actinin 2 modulates host immune responses by inducing tolerogenic dendritic cells via IL-10 production from regulatory T cells. The Korean Journal of Parasitology. 2017 Aug;55(4):375. DOI: [10.3347%2Fkjp.2017.55.4.375](https://dx.doi.org/10.3347%2Fkjp.2017.55.4.375).
- Zhang M, Zheng Y, Sun Y, Li S, Chen L, Jin X, Hou X, Liu X, Chen Q, Li J, Liu M 2019 Knockdown of NEAT1 induces tolerogenic phenotype in dendritic cells by inhibiting activation of NLRP3 inflammasome. Theranostics. 2019;9(12):3425. DOI: [10.7150%2Fthno.33178](https://dx.doi.org/10.7150%2Fthno.33178).
- Perdijk O, Van Neerven RJ, Meijer B, Savelkoul HF, Brugman S 2018 Induction of human tolerogenic dendritic cells by 3′-sialyllactose via TLR4 is explained by LPS contamination. Glycobiology. 2018 Mar 1;28(3):126-30. DOI: [10.1093/glycob/cwx106](https://doi.org/10.1093/glycob/cwx106).
- Li M, Eckl J, Abicht JM, Mayr T, Reichart B, Schendel DJ, Pohla H 2018 Induction of porcine‐specific regulatory T cells with high specificity and expression of IL‐10 and TGF‐β1 using baboon‐derived tolerogenic dendritic cells. Xenotransplantation. 2018 Jan;25(1):e12355. DOI: [10.1111/xen.12355](https://doi.org/10.1111/xen.12355).
- Zhou Y, Leng X, Li H, Yang S, Yang T, Li L, Xiong Y, Zou Q, Liu Y, Wang Y 2017 Tolerogenic dendritic cells induced by BD750 ameliorate proinflammatory T cell responses and experimental autoimmune encephalitis in mice. Molecular Medicine. 2017 Jan;23(1):204-14. DOI: [10.2119/molmed.2016.00110](https://doi.org/10.2119/molmed.2016.00110).
- Eslami‐kaliji F, Sarafbidabad M, Kiani‐Esfahani A, Mirahmadi‐Zare SZ, Dormiani K. 10‐hydroxy‐2‐decenoic acid a bio‐immunomodulator in tissue engineering; generates tolerogenic dendritic cells by blocking the toll‐like receptor 4. Journal of Biomedical Materials Research Part A. 2021 Sep;109(9):1575-87. DOI: [10.1002/jbm.a.37152](https://doi.org/10.1002/jbm.a.37152).
