# Supplementary figures and images for: Tolerogenic dendritic cell reporting: Has a minimum information model made a difference?
Source: PeerJ. 2023 May 31;11:e15352. doi: 10.7717/peerj.15352 (PMC10239229; doi:10.7717/peerj.15352)

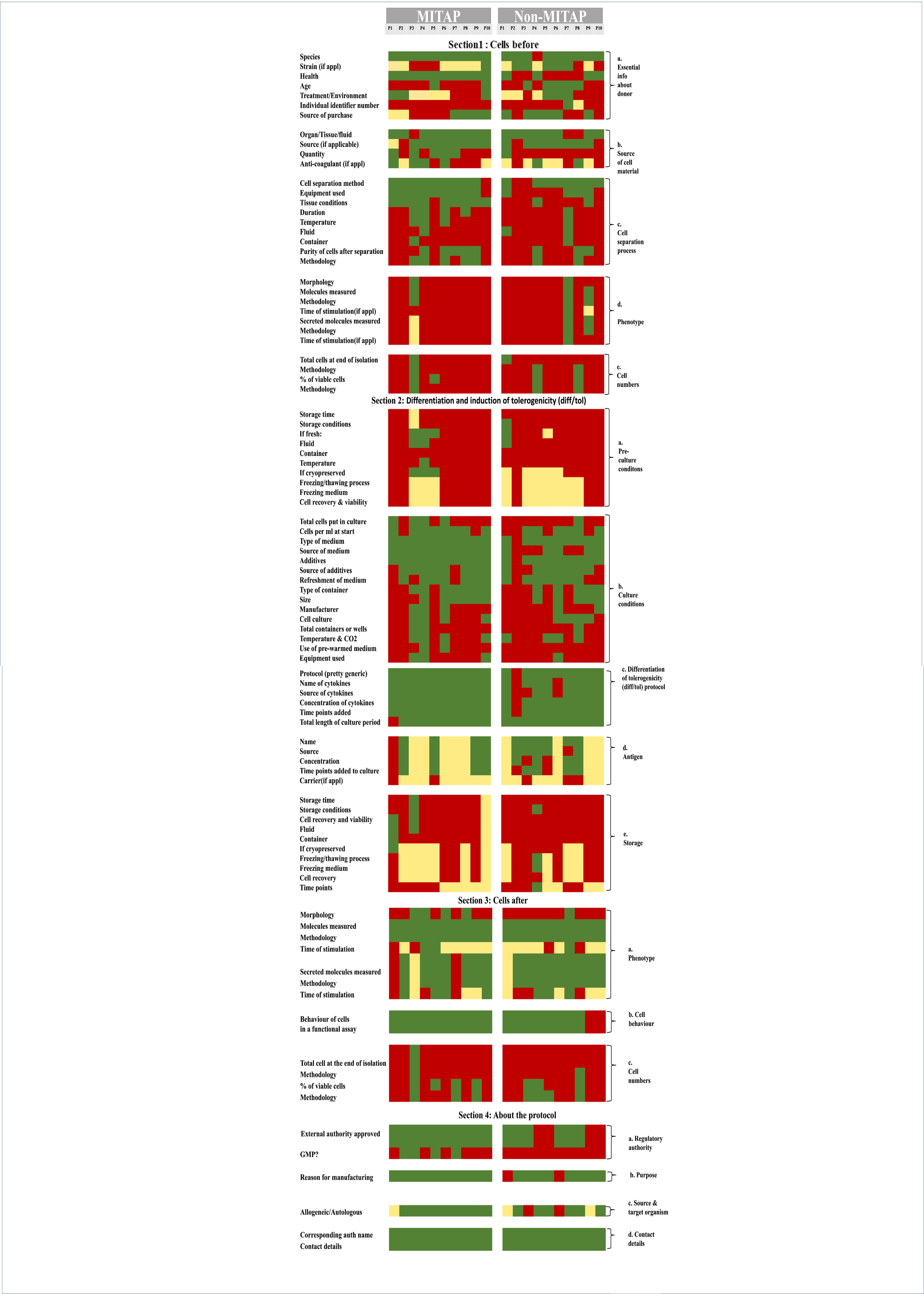

Supplement: Supplemental Information 2 — Green: category reported in the publication; Yellow: category partially reported in the publication; Red circle: category unreported in the publication. [file peerj-11-15352-s002.png]
